# Ezetimibe promotes CYP7A1 and modulates PPARs as a compensatory mechanism in LDL receptor-deficient hamsters

**DOI:** 10.1186/s12944-020-1202-5

**Published:** 2020-02-08

**Authors:** Bin Xia, Ping Lin, Yubin Ji, Jiayu Yin, Jin Wang, Xiaoqian Yang, Ting Li, Zixun Yang, Fahui Li, Shoudong Guo

**Affiliations:** 1 Institute of Lipid Metabolism and Atherosclerosis, Innovative Drug Research Centre, School of Pharmacy, Weifang Medical University, 7166# Baotongxi Street, Weifang, 261053 Shandong Province China; 2 College of Pharmacy Engineering Research Center for Medicine, Harbin University of Commerce, Harbin, 150076 China

**Keywords:** Cholesterol absorption, Lipid metabolism, LDL receptor, Reverse cholesterol transport

## Abstract

**Background:**

The LDL-C lowering effect of ezetimibe has been attributed primarily to increased catabolism of LDL-C via up-regulation of LDL receptor (LDLR) and decreased cholesterol absorption. Recently, ezetimibe has been demonstrated to have reverse cholesterol transport (RCT) promoting effects in mice, hamsters and humans. However, the underlying mechanisms are still not clear. The aim of this study is to investigate whether ezetimibe improves RCT-related protein expression in LDLR^*−/−*^ hamsters.

**Methods:**

A high-fat diet was used to induce a human-like hyperlipidemia in LDLR^*−/−*^ hamsters. Lipid profiles were assayed by commercially available kits, and the effects of ezetimibe on lipid metabolism-related protein expression were carried out via western blot.

**Results:**

Our data demonstrated that ezetimibe administration significantly reduced plasma total cholesterol (~ 51.6% reduction, *P* < 0.01) and triglyceride (from ~ 884.1 mg/dL to ~ 277.3 mg/dL) levels in LDLR^*−/−*^ hamsters fed a high-fat diet. Ezetimibe administration (25 mg/kg/d) significantly promoted the protein expression of cholesterol 7 alpha-hydroxylase A1 (CYP7A1), LXRβ and peroxisome proliferator-activated receptor (PPAR) γ; and down-regulated the protein expression of PPARα and PPARβ. However, it showed no significant effect on sterol regulatory element-binding protein (SREBP)-1c, SREBP-2, proprotein convertase subtilisin/kexin type 9 (PCSK9), Niemann-Pick C1-like 1 (NPC1L1), and ATP-biding cassette (ABC) G5/G8.

**Conclusion:**

Ezetimibe may accelerate the transformation from cholesterol to bile acid via promoting CYP7A1 and thereby enhance RCT. As a compensatory mechanism of TG lowering, ezetimibe promoted the protein expression of PPARγ and decreased PPARα and β. These results are helpful in explaining the lipid-lowering effects of ezetimibe and the potential compensatory mechanisms.

## Background

Hyperlipidemia is an important pathogenic factor of atherosclerosis, which is the basic pathological change of cardiovascular disease [1]. On average, ~ 50% of cholesterol in the gut lumen is reabsorbed in humans and rodents [2, 3], and the remainder excreted in feces. Decreased intestinal absorption of cholesterol is associated with reduced mortality from atherosclerotic cardiovascular disease. Therefore, inhibition of intestinal absorption of cholesterol is a good way to reduce hyperlipidemia and atherosclerotic cardiovascular disease.

Ezetimibe is developed as a cholesterol absorption inhibitor mainly via targeting Niemann-Pick C1-like 1 (NPC1L1) in the intestine [4]. In humans, once-daily treatment with ezetimibe monotherapy (10 mg) inhibits cholesterol absorption on average by 54–65% and results in a ~ 20% reduction of plasma low-density lipoprotein (LDL) cholesterol (LDL-C) [5]. In high-fat fed rhesus monkeys, ezetimibe causes ~ 69% of cholesterol reduction in chylomicrons during the postprandial phase which indirectly lead to a decrease of LDL-C and particle number [6]. It is worthy to note that recent studies suggested that ezetimibe may also promote reverse cholesterol transport (RCT) [7, 8], which plays a key role in cholesterol homeostasis.

RCT is a multi-step process that involves the efflux of cholesterol from peripheral cells to plasma, the hepatic uptake, the biliary excretion, and fecal excretion [9]. Biliary cholesterol can be derived from at least three distinct sources: hepatic de novo cholesterol synthesis, hepatic cholesterol stores, or cholesterol cleared from plasma lipoproteins [10]. The transintestinal cholesterol excretion (TICE) route, independent on biliary route in RCT, for cholesterol excretion in the feces was also provided [11]. Tang et al. viewed all these pathways as a cholesterol transport system [12].

A previous report demonstrated that treatment of C57BL/6 J mice with ezetimibe decreased dietary cholesterol absorption by 86% and increased RCT from peripheral tissue macrophages by 6-fold [13]. Xie et al. suggested that ezetimibe can promote macrophage RCT via inhibition of hepatic NPC1L1 function in transgenic liver-only human NPC1L1 mice [3]. Altemus et al. revealed that the macrophage-to-feces RCT promoting effect of ezetimibe was associated with increased expression of hepatic ABCG5 and ABCG8, which was due to the inhibition of intestinal cholesterol absorption [14]. However, Wang et al. demonstrated that ezetimibe may promote cholesterol elimination via an ABCG5/G8-independent pathway [15]. In hyperlipidemic patients, ezetimibe treatment approximately doubled the flux of plasma-derived cholesterol into fecal neutral sterols, in association with increases in fractional clearance rate of plasma cholesterol ester, and plasma de novo cholesterol synthesis [10]. These studies indicated that ezetimibe may promote the net transfer of cholesterol from peripheral cells for ultimate elimination in the feces. However, some studies have challenged the hypothesis that ezetimibe increases RCT through decreased reabsorption of biliary cholesterol in the intestine [16].

One of the limitations of the previous studies in mice is that mice are high-density lipoprotein cholesterol (HDL-C) dominant, while humans are non-HDL-C dominant [17]. Furthermore, mice are naturally lack cholesteryl ester transfer protein, which is important for cholesterol metabolism. Therefore, the results collected from mice may not reflect the actual situations in humans. Furthermore, the previous results in mice seem to be inconsistent between groups, and the underlying mechanisms need to be further confirmed by other animal models [14, 15]. Although the studies in humans have elucidated some useful phenomena, the underlying mechanisms are not convenient to be carried out in humans. Therefore, it is interesting to study whether ezetimibe improves RCT by improving RCT-related protein expression in animals whose lipid profile is close to that of humans.

Golden Syrian hamster is known to develop human-like hyperlipidemia following a high-fat diet [18]. Additionally, the LDL-C lowering effect of ezetimibe is usually attributed primarily to increased catabolism of LDL-C via up-regulation of LDL receptor (LDLR) [19]. Therefore, it is interesting to investigate whether ezetimibe improves RCT-related protein expression in LDLR^*−/−*^ hamsters fed a high-fat diet, and other potential effects beyond what is presently known.

## Methods

### Materials

Ezetimibe was the product of Selleck (Shanghai, China). High-fat diet (21% fat and 0.25% cholesterol) was provided by Beijing HFK Bioscience Co., Ltd. Complete protease inhibitor cocktail tablets were purchased from Roche (Schweiz, Germany). RIPA lysis buffer was a product of Solarbio (Beijing, China). Rabbit polyclonal antibody against Liver X receptor (α) and LXRβ, and rabbit monoclonal antibody against scavenger receptor B type 1 (SR-B1) and LDLR were from Abcam (Cambridge, MA, USA). Mouse monoclonal antibody against peroxisome proliferator-activated receptor α (PPARα), PPARβ and PPARγ, and cholesterol 7 alpha-hydroxylase A1 (CYP7A1), Niemann-Pick C1-like 1 (NPC1L1), sterol regulatory element-binding protein (SREBP)-1c and SREBP-2 were purchased from Santa Cruz Biotechnology (Santa Cruz, CA, USA). Mouse monoclonal antibody against β-actin and rabbit monoclonal antibody against proprotein convertase subtilisin/kexin type 9 (PCSK9), and rabbit polyclonal antibody against ATP-biding cassette (ABC) G5 were the products of Proteintech (Chicago, IL, USA). Mouse monoclonal antibody against ABCG8 and enhanced chemiluminescence (ECL) kits were purchased from Thermo Scientific Pierce (Rockford, IL, USA). All reagents used in this study were of analytical grade.

### Animals and grouping

Ten LDLR^*−/−*^ Golden Syrian hamsters (male, 165 ± 15 g) were provided by prof. George Liu at Peking University (Beijing, China). All experiments were approved by the Laboratory Animal Ethical Committee of Weifang Medical University and followed the NIH guidelines for the care and use of animals. LDLR^*−/−*^ hamsters were fed a high-fat diet. After a one-week adaptive period, the hamsters were randomly divided into two groups, the model group (0.9% sodium chloride by gavage, *n* = 5), and the ezetimibe group (25 mg/kg/d by gavage, *n* = 5). The hamsters were treated for 4 weeks, then they were sampled.

### Plasma analysis

Total cholesterol (TC) and triglyceride (TG) levels in the plasma were determined enzymatically using commercially available assay kits of Biosino Biotechnology and Science, Inc. (Beijing, China) according to the instructions. The mixed plasma of each group was further separated by fast protein liquid chromatography (FPLC). Briefly, 150 μL plasma was loaded onto a Superose™ 6 10/300 gel chromatography column connected to ÄKTA-FPLC system, and eluted with normal saline at a flow rate of 0.3 mL/min [20]. Eluted fractions (0.5 mL per tube) were collected, and the lipid content was assayed using the same TC and TG kits.

### Protein isolation, electrophoresis, and western blotting

Total proteins from the liver and small intestine were extracted using RIPA lysis buffer with complete protease inhibitor according to the manufacturer’s instructions. Equal amounts of protein (20 μg) were subjected to 6% or 10% SDS-PAGE and transferred onto Polyvinylidene fluoride membranes by electroblotting. After blocking in Tris-buffered saline containing 0.1% Tween-20 and 5% nonfat dry milk for 2 h at room temperature, the membranes were incubated with primary antibodies overnight at 4 °C. After washing 3 times, the membranes were incubated with horseradish peroxidase-conjugated secondary antibodies for 2 h at room temperature. Immunoblots were revealed by enhanced chemiluminescence reaction and images were captured by Clinx ChemiScope 6000 Pro (Shanghai, China). Densitometry analysis was conducted using Image-Pro Plus software version 6.0 (Media Cybernetics Corp, Bethesda, MD, USA) and normalized by housekeeping protein β-actin [21, 22].

### Data analysis

All of the bioassay results were expressed as the mean ± standard deviation (*SD*) for at least three independent experiments. Statistical analysis was performed using one-way analysis of variance (ANOVA) followed by Tukey’s test. Differences were considered to be significant at a *P* < 0.05.

## Results

### Ezetimibe lowered plasma TC and TG levels in LDLR^*−/−*^ hamsters

**Fig. 1:**
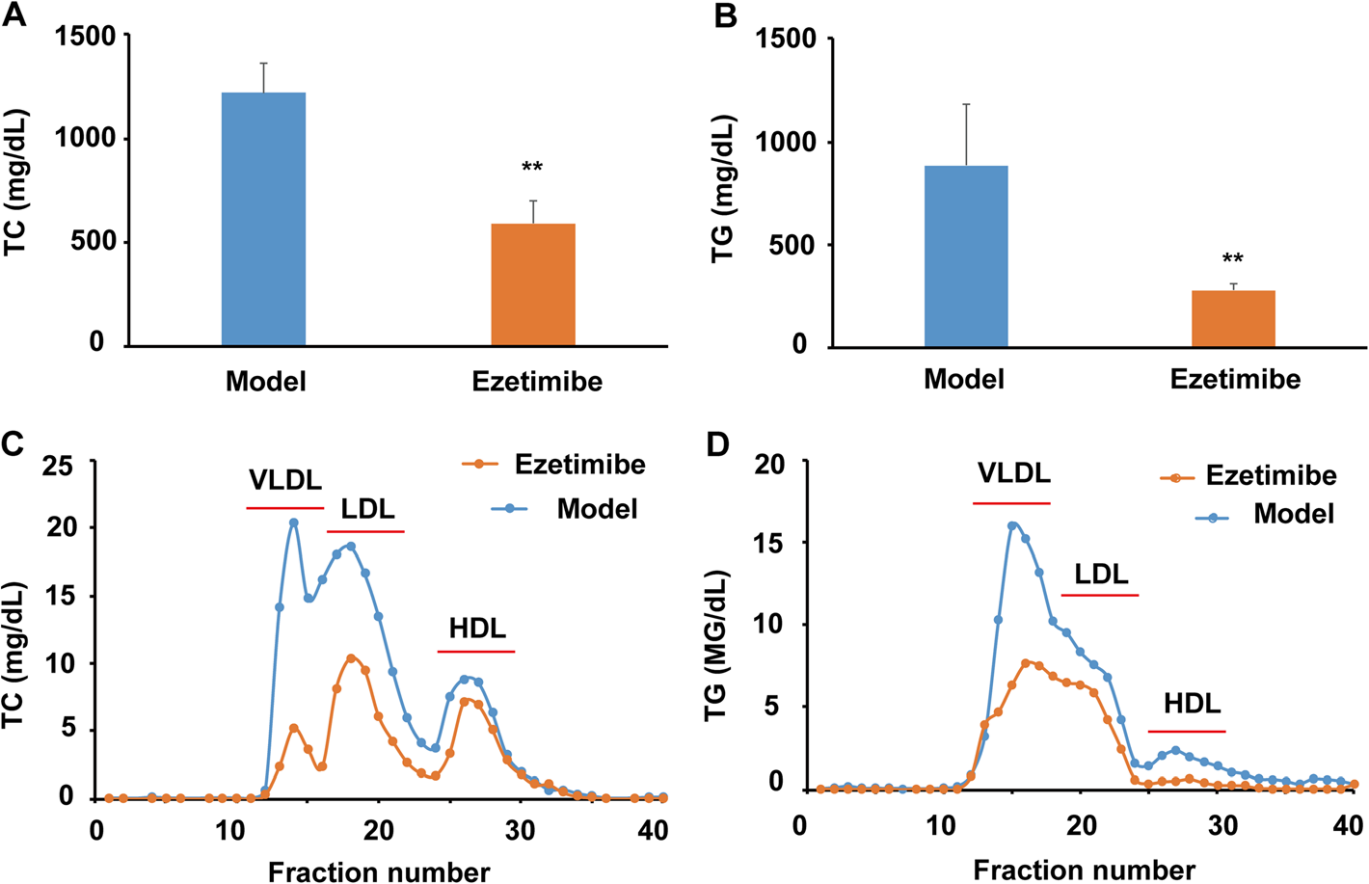
Effect of ezetimibe on the plasma profile of the LDLR^*−/−*^ hamsters fed a high-fat diet (*n* = 5). **a**, ezetimibe lowers plasma TC of the LDLR^*−/−*^ hamsters; **b**, ezetimibe lowers plasma TG of the LDLR^*−/−*^ hamsters; **c**, TC profiles in different lipoprotein fractions after ÄKTA-FPLC separation; **d**, TG profiles in different lipoprotein fractions after ÄKTA-FPLC separation. Data are expressed as mean ± SD. ^**^p < 0.01 vs model group

As shown in Fig. 1a, ezetimibe administration significantly reduced plasma TC of the LDLR^*−/−*^ hamsters fed a high-fat diet when compared with the model group (~ 51.6% reduction, *P* < 0.01). Furthermore, ezetimibe treatment lowered the TG levels of the LDLR^*−/−*^ hamsters from ~ 884.1 mg/dL to ~ 277.3 mg/dL (Fig. 1b, ~ 68.6% reduction, *P* < 0.01). Further lipid profile analysis after ÄKTA-FPLC separation indicated that ezetimibe treatment significantly decreased the cholesterol levels of very low-density lipoprotein (VLDL) and LDL particles, and exhibited comparably lower effect on HDL-C (Fig. 1c). Furthermore, ezetimibe administration also significantly lowered the TG content of VLDL, LDL, and HDL particles (Fig. 1d).

### Ezetimibe improved CYP7A1 expression in the liver of the LDLR^*−/−*^ hamsters

**Fig. 2:**
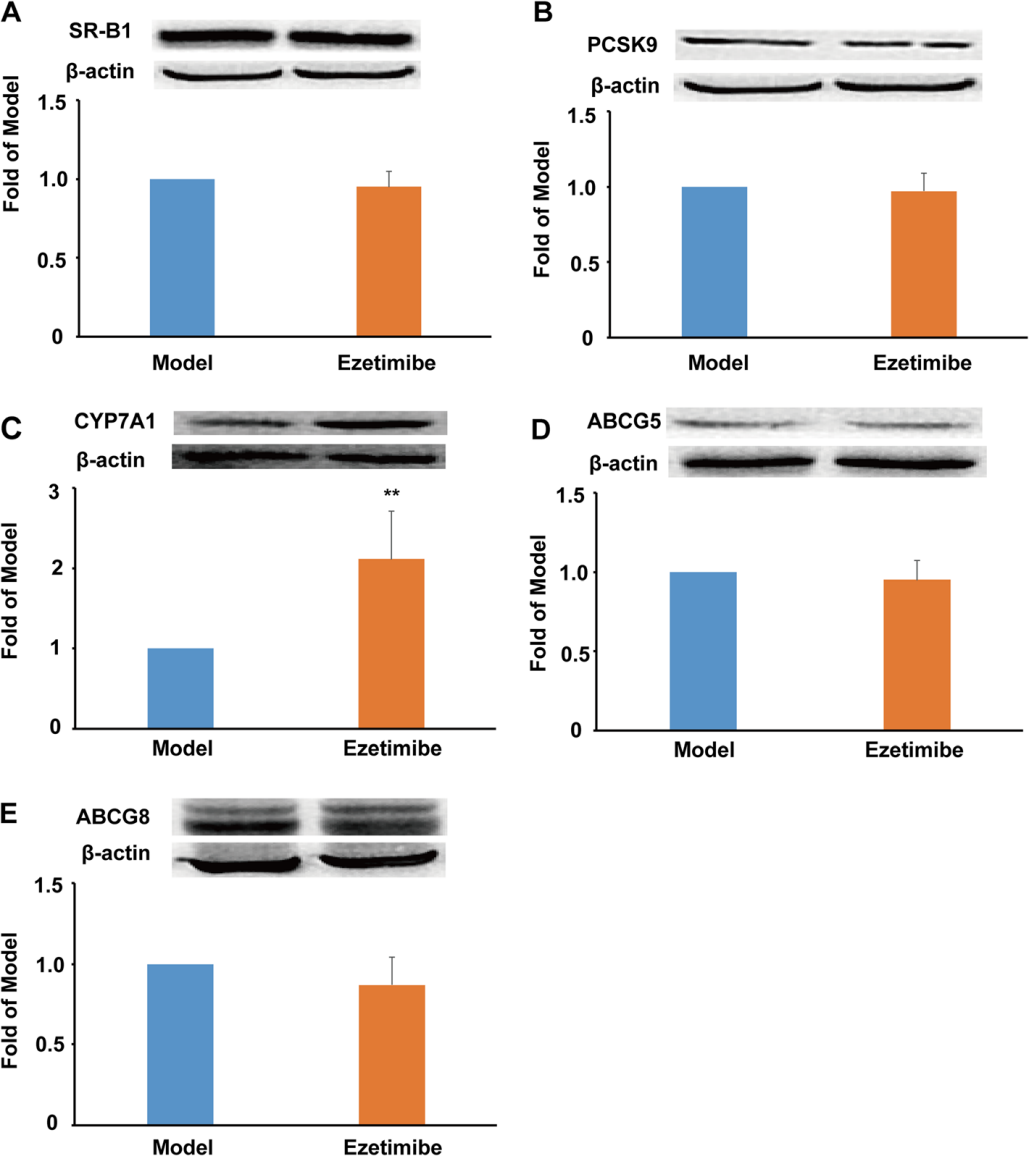
Effect of ezetimibe on the RCT-related protein expression in the liver of the LDLR^*−/−*^ hamsters (*n* = 3). **a**, protein expression of SR-B1 and densitometric quantification; **b**, protein expression of PCSK9 and densitometric quantification; **c**, protein expression of CYP7A1 and densitometric quantification; **d**, protein expression of ABCG5 and densitometric quantification; **e**, protein expression of ABCG8 and densitometric quantification. Data are expressed as mean ± SD. ^**^p < 0.01 vs model group

In this study, ezetimibe treatment showed no significant effect on the protein expression of SR-B1 (Fig. 2a), which plays a key role in hepatic uptake of HDL-C [9, 12]. LDLR delivers non-HDL particles to the liver, and PCSK9 binds LDLR and leads to its degradation in the endosome [23]. In LDLR^***−/−***^ hamsters, the protein expression of LDLR was not detectable (data not shown), and ezetimibe administration exhibited no significant effect on the protein expression of PCSK9 in this study (Fig. 2b). CYP7A1 is the first rate-limiting enzyme of bile acid synthesis. It is worthy to note that ezetimibe treatment significantly promoted the protein expression of CYP7A1 compared to the model group (~ 2.1-fold, *P* < 0.01, Fig. 2c). However, ezetimibe treatment showed no significant effect on the protein expression of ABCG5 and ABCG8 (Fig. 2d and e), which play key roles in the transport of bile acid and cholesterol to the gall bladder for excretion.

### Ezetimibe down-regulated PPARα and β and up-regulated PPARγ expression in the liver of the LDLR^*−/−*^ hamsters

**Fig. 3:**
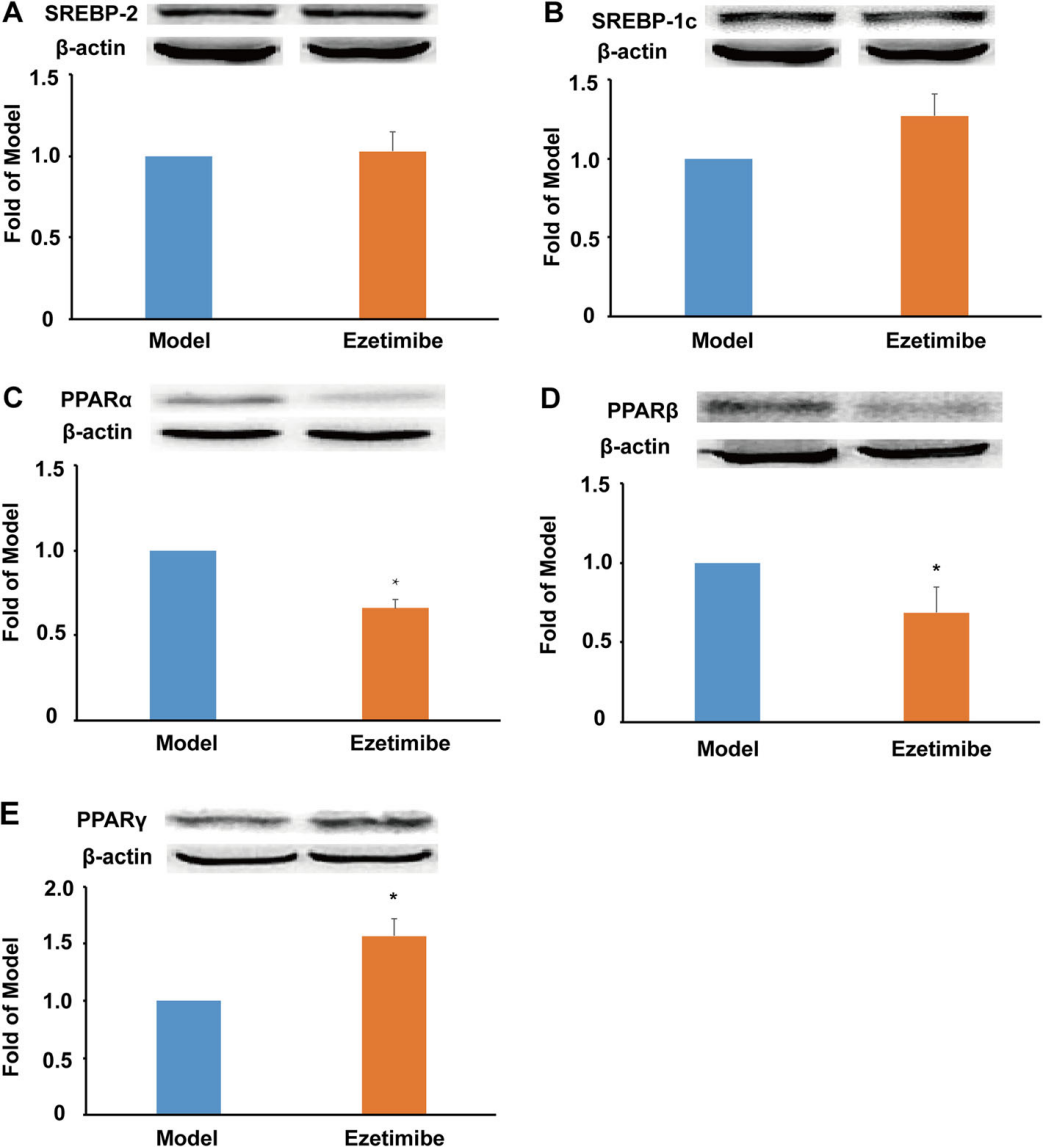
Effect of ezetimibe on the protein expression of SREBPs and PPARs in the liver of the LDLR^*−/−*^ hamsters (*n* = 3). **a**, protein expression of SREBP-2 and densitometric quantification; **b**, protein expression of SREBP-1c and densitometric quantification; **c**, protein expression of PPARα and densitometric quantification; **d**, protein expression of PPARβ and densitometric quantification; **e**, protein expression of PPARγ and densitometric quantification. ^*^p < 0.05 vs model group

SREBPs are important transcription factors involved in the regulation of lipid metabolism and homeostasis. In the present study, ezetimibe showed no significant influence on the protein expression of both SREBP-2 and SREBP-1c (Fig. 3a and b). PPARs are key regulators of lipid storage, especially TG metabolism and utilization of fat. In contrast to the model group, ezetimibe treatment significantly reduced the protein expression of both PPARα and β (~ 32.5%, *P* < 0.05, Fig. 3c and d). Furthermore, ezetimibe administration significantly promoted the protein expression of PPARγ compared with the model group (~ 1.5-fold, *P* < 0.05, Fig. 3e).

### Ezetimibe up-regulated LXRβ expression in the small intestine of the LDLR^*−/−*^ hamsters

**Fig. 4:**
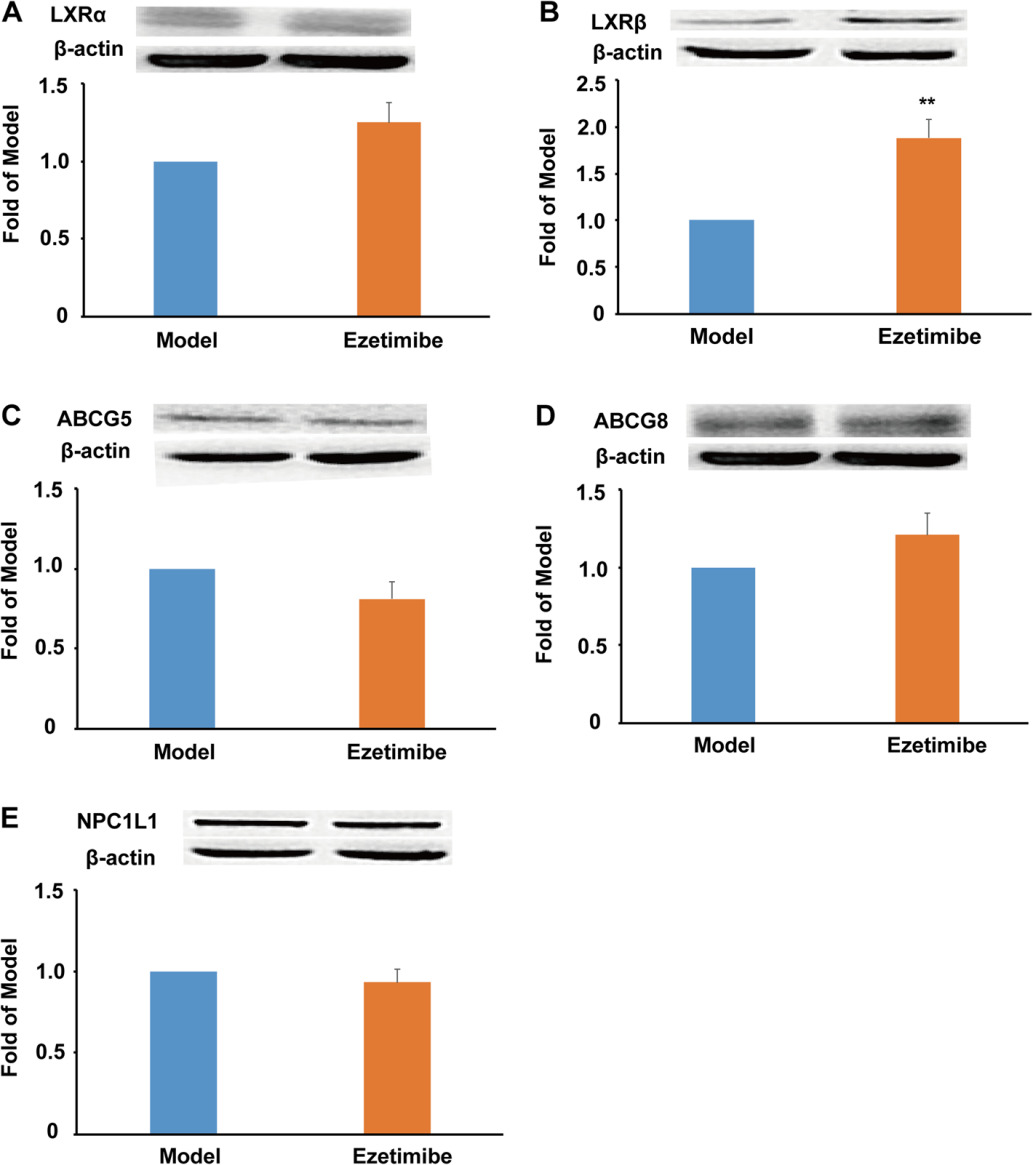
Effect of ezetimibe on the protein expression of LXR/ABC transporters and NPC1L1 in the small intestine of the LDLR^*−/−*^ hamsters (*n* = 3). **a**, protein expression of LXRα and densitometric quantification; **b**, protein expression of LXRβ and densitometric quantification; **c**, protein expression of ABCG5 and densitometric quantification; **d**, protein expression of ABCG8 and densitometric quantification; **e**, protein expression of NPC1L1 and densitometric quantification. ^**^p < 0.01 vs model group

LXRs regulate genes that encode proteins involved in lipid transport and excretion. In this study, ezetimibe showed no significant effect on the protein expression of LXRα (Fig. 4a). It is worthy to note that ezetimibe dramatically promoted the protein expression of LXRβ when compared to the model group (~ 1.8-fold, *P* < 0.01, Fig. 4b). However, ezetimibe treatment showed no significant effect on the protein expression of ABCG5 and ABCG8 (Fig. 4c and d). Furthermore, ezetimibe showed no effect on the protein expression of NPC1L1 compared with the model group (Fig. 4e). This result further suggested that ezetimibe inhibits the function rather than the protein expression of NPC1L1.

## Discussion

Elevated cholesterol is an important risk factor for atherosclerotic cardiovascular disease, and strategies to reduce cholesterol levels have been used to reduce the risk of atherosclerotic cardiovascular disease. Ezetimibe, a clinically used cholesterol absorption inhibitor, works via inhibiting the function of NPC1L1 [4]. In this LDLR^*−/−*^ hamster study, we report for the first time that: 1) ezetimibe significantly promoted the protein expression of CYP7A1 and PPARγ, and lowered the protein expression of PPARα and PPARβ in the liver; 2) ezetimibe significantly up-regulated the protein expression of LXRβ in the small intestine, but not NPC1L1 and ABCG5/G8; and 3) ezetimibe may not influence PCSK9 in the absent of LDLR. Given that this study was performed in the animal model lacking LDLR, these data may be more relevant for humans with familial hypercholesterolemia than for general population.

As previously reported by Guo et al., high-fat diet can induce human-like hyperlipidemia in LDLR^*−/−*^ hamsters [17]. The TC-lowering effect of ezetimibe was consistent with the results obtained from C57BL/6 J mice [13], apolipoprotein E knockout mice [24], rhesus monkeys [6], and humans [10]. The mechanisms may be attributed to the significant decreased dietary cholesterol absorption (~ 86% in C57BL/6 J mice) and the increased RCT from peripheral tissue macrophages [13]. The TG-lowering effect of ezetimibe in LDLR^*−/−*^ hamsters was inconsistent with the data in rhesus monkeys [6], and humans [10]. However, our data were consistent with the previous results obtained in LDLR^*−/−*^ animals [17, 19], which also indicated that ezetimibe not only significantly reduced plasma TC levels but also decreased plasma TG levels. These differences may be attributed to the distinct animal models, and especially the absent of LDLR. Given that ezetimibe dose not block fat absorption [25], the activity of ezetimibe in decreasing TG is likely a secondary effect of diminished cholesterol delivery in LDLR^*−/−*^ mice or hamsters [19, 26]. Due to the significant difference in lipid profile between the ezetimibe and model group and especially the small variation within each group, 3 of the 5 hamsters per group were randomly chosen for the immunoblotting experiments in the present study.

The LDL-C lowering effect of ezetimibe is usually attributed primarily to increased catabolism of LDL-C via up-regulation of LDLR [19, 27]. The changes of LDL-C in LDLR^*−/−*^ models can directly refect the rate of LDL-C production. Our data together with the previous publication [19] support that ezetimibe treatment can decrease LDL production. Given the absence of LDLR in LDLR^*−/−*^ hamsters, SR-B1 plays a more important role in the hepatic uptake of cholesterol from plasma. However, our data showed that ezetimibe had no effect on the expression of SR-B1, which was consistent with a previous study in LDLR^*−/−*^ mice [19]. Additionally, we report here that ezetimibe treatment has no modulatory effect on the protein expression of PCSK9, which functions as a negatively modulator of LDLR [23]. Cholesterol in the liver can be transformed to bile acid and then excreted into bile or secreted back into plasma within VLDL. CYP7A1 is a key rate-limiting enzyme for the transformation from cholesterol to bile acid. In a previous study, ezetimibe seemed to increase the mRNA expression of CYP7A1 based on the treatment of ursodiol in mice [15] and in germ-free or specific pathogen-free mice [28]. In this study, we report for the first time that ezetimibe significantly increased the protein expression of CYP7A1 in LDLR^*−/−*^ hamsters. This result suggested that ezetimibe may accelerate RCT by enhancing the transformation from cholesterol to bile acid. A previous study has successfully demonstrated that ezetimibe enhanced RCT via the hepato-biliary pathway in hamsters [29].

Excretion of cholesterol by the liver and intestine is mediated by the tissue-specific expression of the transporters ABCG5 and ABCG8, which transfer these sterols into the lumen of the biliary tree or intestine, respectively [14, 30]. The ABCG5/G8 heterodimer account for ~ 70 to 90% of biliary cholesterol secretion [31]. In addition to its known suppression of intestinal cholesterol absorption, one previous study demonstrated that ezetimibe can stimulate macrophage-to-feces RCT by indirectly increasing liver ABCG5/G8 mRNA expression, but not in the small intestine [14]. However, another study indicated that ezetimibe increased fecal neutral sterol elimination via ABCG5/G8 independent manner as verified in ABCG5/G8-deficient mice [15]. Furthermore, transgenic mice expressing human NPC1L1 in hepatocytes resulted in 10- to 20-fold decrease in biliary cholesterol concentration, but it showed no effect on the protein expression of ABCG5/G8, SR-B1 and LDLR [32]. In a diet-induced hamster model of insulin resistance, ezetimibe also showed no effect on the protein expression of ABCG5 and ABCG8 [33]. Our data were consistent with the previous studies, which showed ezetimibe treatment has no significant effects on the hepatic and intestinal ABCG5/G8 protein expression. LXRα is the best-studied regulator of ABCG5/G8 expression, however, our results in LDLR^*−/−*^ hamsters and the previous study in C57BL/6 J mice [14] all demonstrated that ezetimibe treatment may not activate LXRα. SREBP-2 is also a regulator of ABCG5 expression in the liver [34], and our data suggested that ezetimibe showed no effect on SREBP-2. Furthermore, the loss of LXRβ seems to have no effect on the expression of ABCG5/G8 [35]. This may explain why there was a significant upregulation of LXRβ, but the protein expression of ABCG5/G8 showed no significant difference in the small intestine of the LDLR^*−/−*^ hamsters.

In humans, ezetimibe treatment enhanced de novo cholesterol synthesis to maintain cholesterol balance. SREBP-2 can activate a series of genes involved in the synthesis of cholesterol, while SREBP-1c plays a key role in TG synthesis. Our data showed that ezetimibe administration had no significant effect on these two important lipid synthesis modulators. Furthermore, PPARα is highly expressed in the liver and its activation can promote the utilization of fat, while PPARγ is a key regulator of adipogenesis [21, 22]. Our data suggested that ezetimibe may enhance TG storage by reducing PPARα and promoting PPARγ expression in response to the significant reduction of TG in ezetimibe-treated LDLR^*−/−*^ hamsters. These kinds of compensatory effects are also found in other ezetimibe administration studies, for example, the up-regulation of hydroxymethylglutaryl-CoA reductase (HMG-CoA) reductase and HMG-CoA synthase [19, 27]. However, ezetimibe showed no effect on the mRNA expression of HMG-CoA reductase and HMG-CoA synthase in C57BL/6 mice [15]. Therefore, the compensatory effects of ezetimibe seem to be different based on distinct animal species.

Finally, it is worthy to note that ezetimibe showed no effect on intestinal NPC1L1 in this study, and the result was consistent with a previous study in high-fat diet hamster [32]. In humans, only about 25–35% of intestinal cholesterol is derived from diet, while the remaining originates from the liver in bile, and perhaps a direct transintestinal cholesterol efflux (TICE) route [11, 12]. Presently, there are still arguments about the role of TICE pathway in promoting macrophage RCT [3]. However, accumulating evidence support that the absorption of cholesterol from the intestine is also an important determinant of macrophage-to-feces RCT [13, 36]. A recent study indicated that TICE may contribute minor to the macrophage-to-feces RCT in hamsters [29]. Unlike humans, hamsters express comparably lower NPC1L1 in the liver [37] as that of mice, which is a limitation of this animal model.

## Conclusions

Ezetimibe significantly lowers TC and TG in LDLR^*−/−*^ hamsters. It may improve RCT by up-regulating CYP7A1, but not ABCG5/G8. As a compensatory mechanism, it down-regulates PPARα and β and up-regulates PPARγ. However, these results need to be confirmed by other animal models.

## Data Availability

The authors made reproducible materials described in the manuscript, freely available to any scientist wishing to use them, without breaching participant confidentiality. The date and methods have been fully presented in the manuscript.

## Abbreviations

ABC: ATP-binding cassettes;
CYP7A1: Cholesterol 7 alpha-hydroxylase A1
HDL: High density lipoprotein
LDL: Low density lipoprotein
NPC1L1: Niemann-Pick C1-like 1
PCSK9: Proprotein convertase subtilisin/kexin type 9
PPAR: Proliferator-activated receptor
RCT: Reverse cholesterol transport
SR-BI: Scavenger receptor B type 1
SREBP: Sterol regulatory element-binding protein
